# Supplementary figures and images for: Performance of modeling and balancing approach methods when using weights to estimate treatment effects in observational time-to-event settings
Source: PLoS One. 2023 Dec 7;18(12):e0289316. doi: 10.1371/journal.pone.0289316 (PMC10703278; doi:10.1371/journal.pone.0289316)

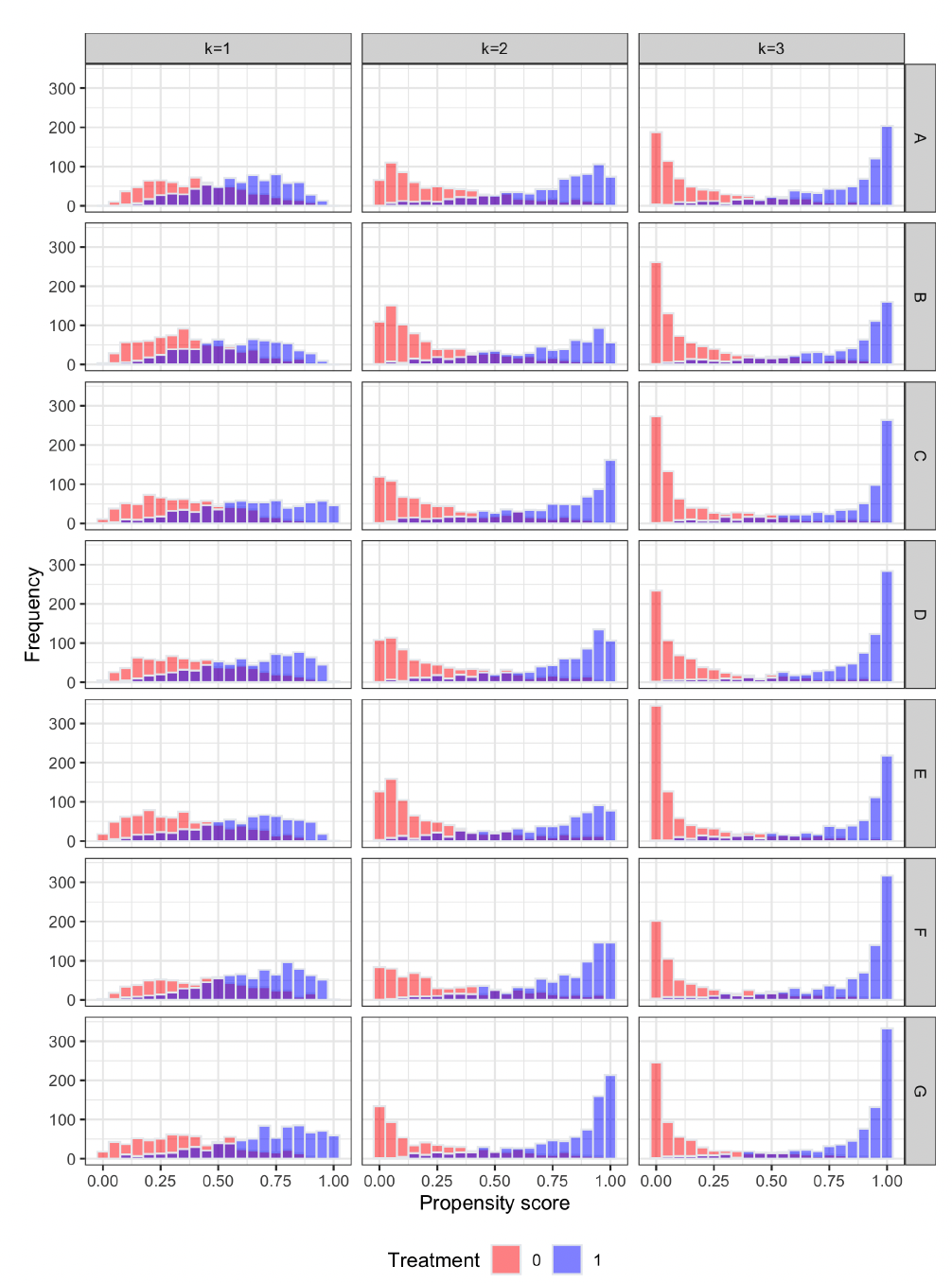

Supplement: S1 Fig — (TIF) [file pone.0289316.s001.tif]

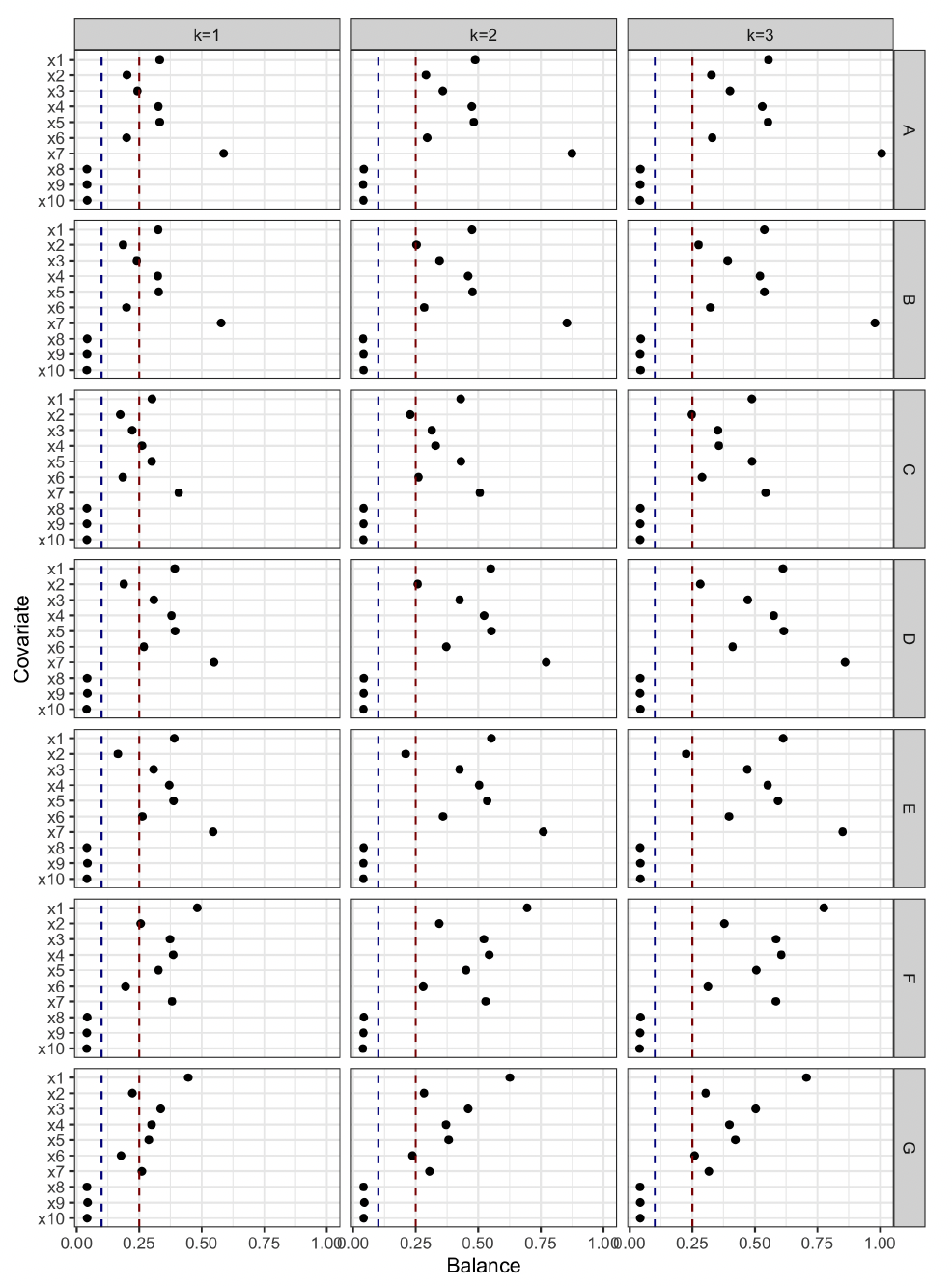

Supplement: S2 Fig — The blue and red dashed lines represents average balance (ASMD or AUD) equal to 0.10 and 0.25, respectively. (TIF) [file pone.0289316.s002.tif]

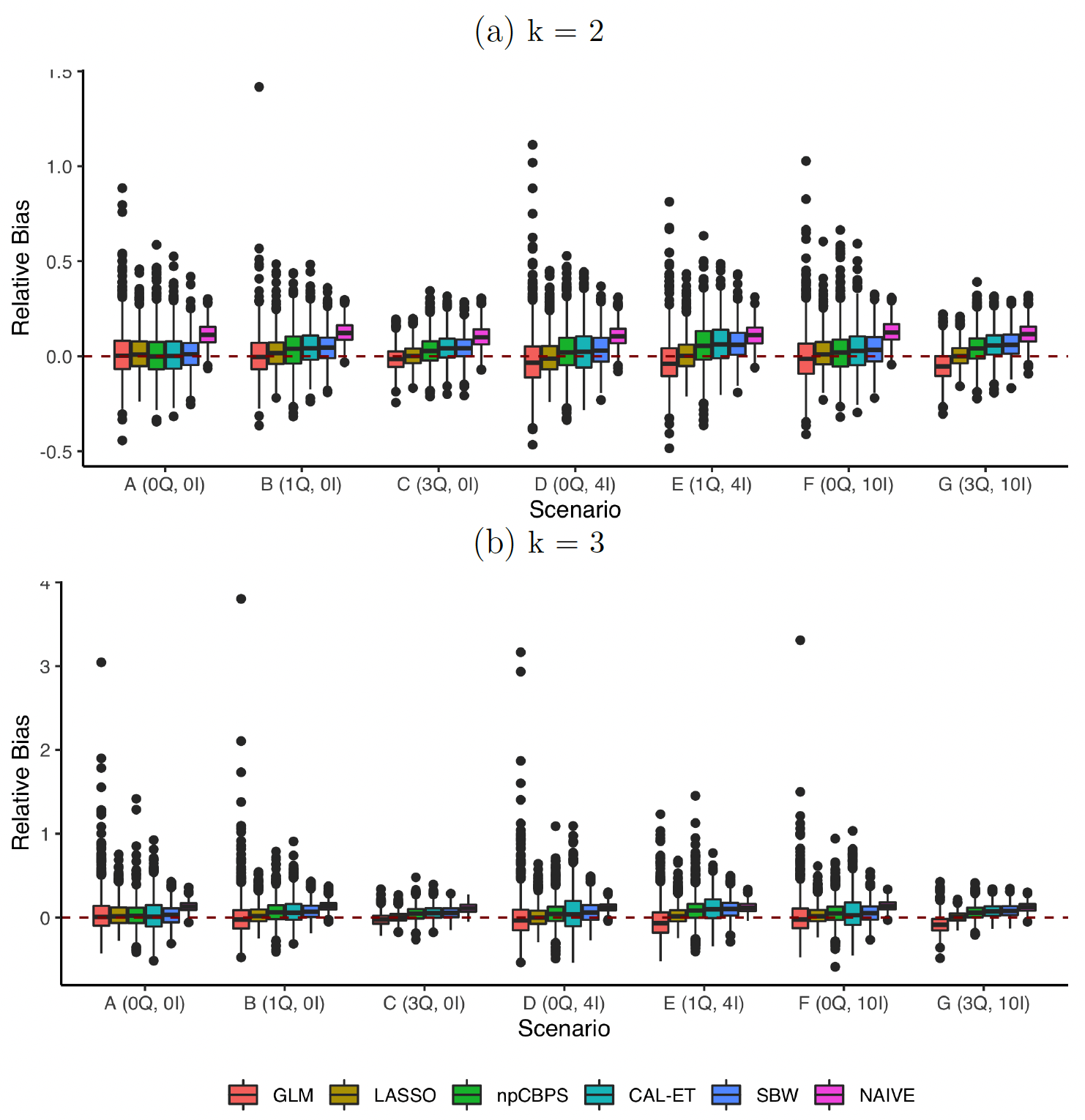

Supplement: S3 Fig — Relative bias of the MHRATT estimation methods for varying degrees of overlap and different DGPs. Sample size is n = 1500, true MHRATT = 0.8 and no censoring (π = 0). In Scenario (A) there is no model misspecification. All outliers are included. (TIF) [file pone.0289316.s003.tif]

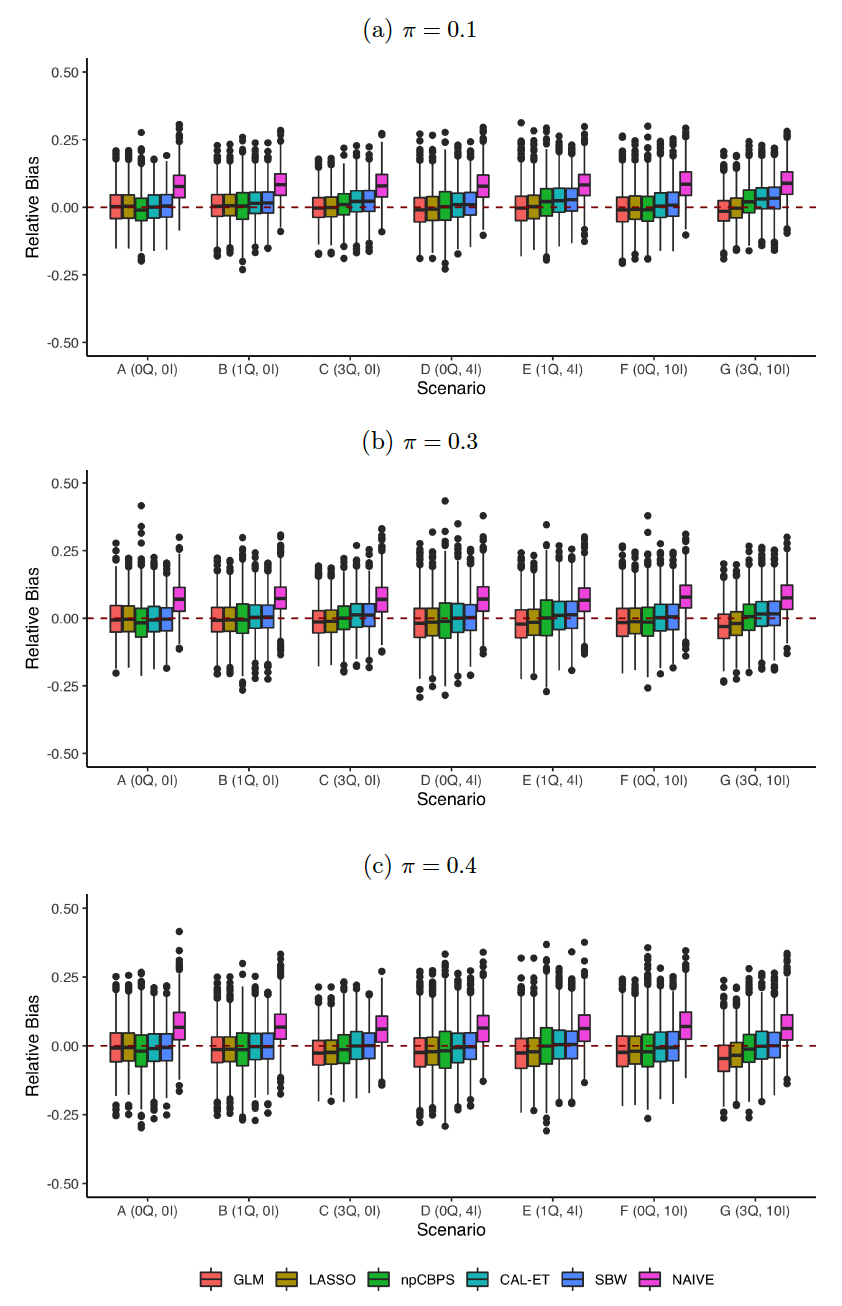

Supplement: S4 Fig — Relative bias of the MHRATT estimation methods for varying censoring rates and different DGPs. Sample size is n = 1500, true MHRATT = 0.8 and good overlap (k = 1). In Scenario (A) there is no model misspecification. (TIF) [file pone.0289316.s004.tif]

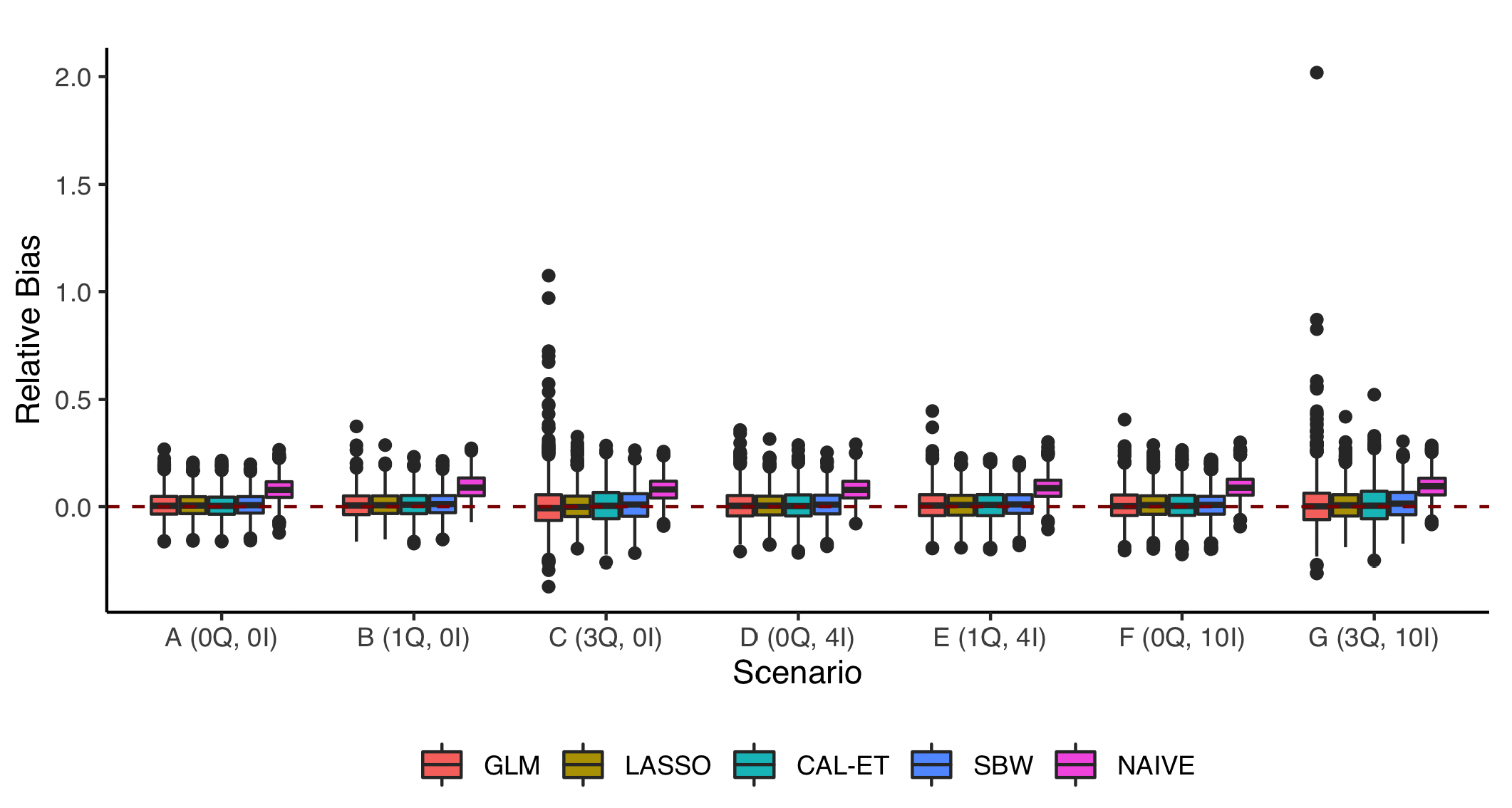

Supplement: S5 Fig — Relative bias of the MHRATT estimation methods when all models are overspecified. Sample size is n = 1500, true MHRATT = 0.8, no censoring (π = 0) and good overlap (k = 1). All outliers are included. (TIF) [file pone.0289316.s005.tif]

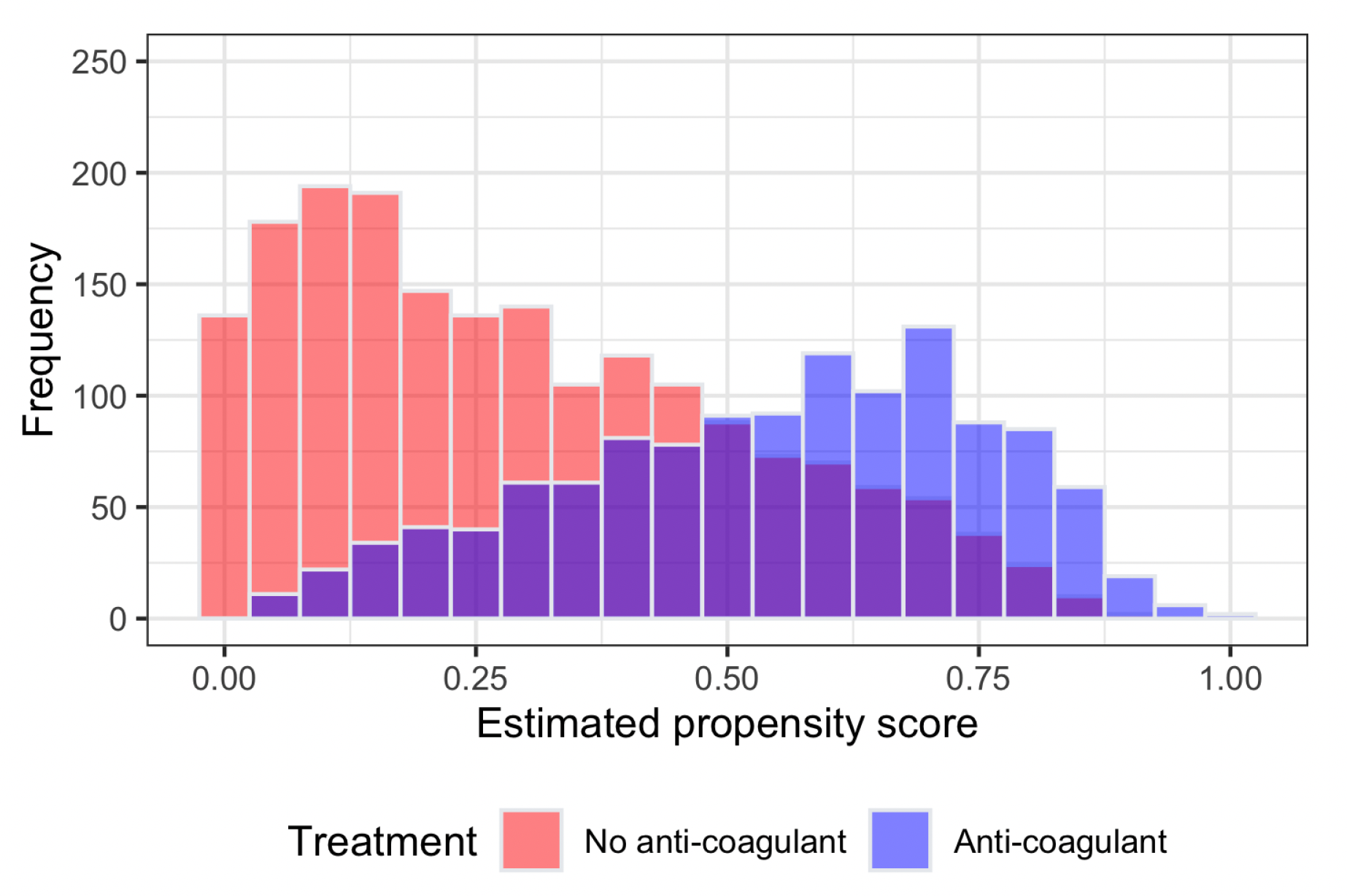

Supplement: S6 Fig — The propensity score is estimated using GLM with Xlarge. (TIF) [file pone.0289316.s006.tif]
