# Supplementary material for: Performance of modeling and balancing approach methods when using weights to estimate treatment effects in observational time-to-event settings
Source: PLoS One. 2023 Dec 7;18(12):e0289316. doi: 10.1371/journal.pone.0289316 (PMC10703278; doi:10.1371/journal.pone.0289316)
Supplement: S1 File — (ZIP) [file pone.0289316.s007.zip › S1_File.pdf]

Performance of modeling and balancing approach methods when using weights to estimate treatment effects in observational time-to-event settings — Supporting information

Guilherme W. F. Barros*[1], Marie Eriksson[1], and Jenny Häggström[1]

[1]Department of Statistics, Umeå School of Business, Economics and Statistics, Umeå University, Umeå, Sweden

A S1 Appendix: Generating Censored Data

When generating censored data, in addition to the survival time Y, the censoring time C must be generated. C is often assumed to follow a Weibull(η, θ) or Uniform(0, θ) distribution, but the value for θ must be decided upon by the researcher. Wan (2016) [1] presented a general framework to generate censoring times with predefined censoring rates for proportional hazard models, i.e., a method to find the value of θ such that a certain proportion of the generated data is right-censored. This is done by solving the integral

$$\gamma(\theta|\pi) = \int_{D'} \Pr(\omega = 1|u, \theta) f_{\tau_i}(u) du - \pi,$$

where π is the censoring proportion, D' is the domain of $\tau_i = \frac{\exp(LP_i/\eta)}{\sqrt{\lambda}}$, $\omega = I(Y \geq C)$ is the censoring indicator and $f_{\tau_i}(\cdot)$ is the density function of τ_i. If $C \sim$ Uniform$(0, \theta)$, the individual censoring probability can be expressed as

$$\Pr(\omega = 1|\tau_i, \eta, \theta) = \frac{\tau_i}{\eta\theta} \Gamma\left(\frac{1}{\eta}, (\theta/\tau_i)^\eta\right),$$

where $\Gamma(\cdot, \cdot)$ is the lower incomplete gamma function.

In our scenarios, as the covariates $\mathbf{X}$ come from different distributions, it is not possible to derive $f_{\tau_i}(u)$ explicitly. Therefore, we, as suggested by Wan (2016) [1] estimate it using a kernel smoothing method. For that, we use a Gaussian kernel,

$$K(u) = \frac{1}{\sqrt{2\pi}} e^{-u^2/2},$$

and a scaled kernel is constructed at every data point u_i

$$h^{-1}K((u - u_i)/h).$$

Here, h is the smoothing parameter that govern the bias-variance tradeoff. High values of h result in higher bias and lower variance, while lower values give lower bias but higher variance. We use Silverman's rule of thumb [2] for selecting h,

$$\hat{h} = 1.06 \min(\hat{\sigma}, \text{IQR}/1.34) n^{-1/5},$$

***Correspondence** Department of Statistics, Umeå School of Business, Economics and Statistics, Umeå University, SE-901 87 Umeå, Sweden. Email: guilherme.barros@umu.se

where $\hat{\sigma}$ and IQR are the sample standard deviation and interquartile range of τ_i, respectively. Finally, we can estimate the density at a new point u by taking the mean of all individual scaled kernel functions

$$\hat{f}_{\tau_i}(u) = \frac{1}{nh}\sum_{i=1}^{n} K((u-u_i)/h).$$

We used the `density` function in the `R` package `stats` to estimate the density as described above.

B S2 Appendix: Simulation study results

Figure S1: Propensity score overlap based on one replication of simulated data ($n = 1500$) for each scenario and degree of overlap.

Figure S2: Covariate balance averaged over 1000 replicates of simulated data ($n = 1500$) for each scenario and degree of overlap. The blue and red dashed lines represents average balance (ASMD or AUD) equal to 0.10 and 0.25, respectively.

Figure S3: *Experiment 1: Misspecification and Overlap.* Relative bias of the MHR_{ATT} estimation methods for varying degrees of overlap and different DGPs. Sample size is $n = 1500$, true $\mathrm{MHR}_{ATT} = 0.8$ and no censoring ($\pi = 0$). In scenario (A) there is no model misspecification. All outliers are included.

Figure S4: *Experiment 2: Misspecification and Censoring.* Relative bias of the MHR_{ATT} estimation methods for varying censoring rates and different DGPs. Sample size is $n = 1500$, true $\text{MHR}_{ATT} = 0.8$ and good overlap ($k = 1$). In scenario (A) there is no model misspecification.

Figure S5: *Experiment 4: Overspecification.* Relative bias of the MHR_{ATT} estimation methods when all models are overspecified. Sample size is $n = 1500$, true $\text{MHR}_{ATT} = 0.8$, no censoring ($\pi = 0$) and good overlap ($k = 1$). All outliers are included.

Table S1: Results of 1000 simulation replicates for estimators of MHR_{ATT} for different DGPs (A) - (G). Bias, Monte Carlo standard deviation (SD), root mean-squared error (RMSE), empirical coverage probability of 95% confidence intervals (Coverage and relative bias (Rel.Bias). Sample size is $n = 1500$, true $\mathrm{MHR}_{ATT} = 0.8$, no censoring ($\pi = 0$) and good overlap ($k = 1$). In scenario (A) there is no model misspecification.

Method	Scenario	Bias	SD	RMSE	Coverage	Rel. Bias
GLM	(A)	0.004	0.050	0.050	0.965	0.005
LASSO		0.005	0.048	0.049	0.966	0.007
npCBPS		-0.005	0.054	0.054	0.962	-0.006
CAL-ET		0.004	0.048	0.048	0.976	0.004
SBW		0.004	0.046	0.046	0.975	0.005
NAIVE		0.063	0.045	0.078	0.715	0.079
GLM	(B)	0.009	0.048	0.049	0.970	0.011
LASSO		0.010	0.047	0.048	0.967	0.013
npCBPS		0.009	0.054	0.055	0.964	0.011
CAL-ET		0.017	0.047	0.050	0.966	0.022
SBW		0.017	0.046	0.049	0.963	0.022
NAIVE		0.070	0.046	0.084	0.642	0.087
GLM	(C)	0.002	0.041	0.041	0.965	0.002
LASSO		0.004	0.040	0.040	0.967	0.005
npCBPS		0.013	0.045	0.047	0.968	0.016
CAL-ET		0.021	0.044	0.048	0.966	0.027
SBW		0.022	0.042	0.048	0.963	0.028
NAIVE		0.066	0.045	0.080	0.662	0.083
GLM	(D)	0.000	0.054	0.054	0.963	0.000
LASSO		0.003	0.052	0.052	0.966	0.004
npCBPS		0.006	0.060	0.060	0.972	0.007
CAL-ET		0.014	0.053	0.055	0.972	0.018
SBW		0.016	0.051	0.053	0.956	0.020
NAIVE		0.067	0.046	0.082	0.659	0.084
GLM	(E)	0.003	0.051	0.051	0.965	0.003
LASSO		0.007	0.049	0.050	0.967	0.009
npCBPS		0.018	0.062	0.064	0.950	0.023
CAL-ET		0.026	0.051	0.057	0.963	0.032
SBW		0.026	0.048	0.055	0.964	0.032
NAIVE		0.069	0.046	0.084	0.639	0.087
GLM	(F)	-0.001	0.052	0.052	0.968	-0.001
LASSO		0.003	0.049	0.049	0.967	0.004
npCBPS		0.003	0.055	0.055	0.966	0.004
CAL-ET		0.011	0.050	0.051	0.970	0.014
SBW		0.013	0.049	0.050	0.967	0.017
NAIVE		0.075	0.044	0.087	0.623	0.094
GLM	(G)	-0.006	0.046	0.046	0.967	-0.008
LASSO		0.002	0.044	0.044	0.968	0.003
npCBPS		0.019	0.050	0.053	0.955	0.024
CAL-ET		0.029	0.046	0.055	0.952	0.036
SBW		0.031	0.046	0.055	0.947	0.039
NAIVE		0.076	0.048	0.090	0.586	0.096

Table S2: Moderate overlap ($k = 2$). Results of 1000 simulation replicates for estimators of MHR_{ATT} for different DGPs (A) - (G). Bias, Monte Carlo standard deviation (SD), root mean-squared error (RMSE), empirical coverage probability of 95% confidence intervals (Coverage and relative bias (Rel.Bias). Sample size is $n = 1500$, true $\text{MHR}_{ATT} = 0.8$ and no censoring ($\pi = 0$). In scenario (A) there is no model misspecification.

Method	Scenario	Bias	SD	RMSE	Coverage	Rel. Bias
GLM	(A)	0.010	0.104	0.104	0.946	0.012
LASSO		0.014	0.080	0.081	0.955	0.017
npCBPS		0.004	0.092	0.092	0.961	0.005
CAL-ET		0.008	0.086	0.086	0.956	0.010
SBW		0.011	0.069	0.070	0.964	0.014
NAIVE		0.091	0.048	0.103	0.476	0.114
GLM	(B)	0.004	0.094	0.094	0.958	0.006
LASSO		0.017	0.070	0.072	0.972	0.021
npCBPS		0.029	0.089	0.093	0.939	0.036
CAL-ET		0.038	0.075	0.084	0.976	0.048
SBW		0.038	0.063	0.073	0.963	0.048
NAIVE		0.100	0.045	0.110	0.392	0.125
GLM	(C)	-0.012	0.050	0.051	0.959	-0.014
LASSO		0.002	0.046	0.046	0.966	0.003
npCBPS		0.023	0.063	0.067	0.962	0.029
CAL-ET		0.035	0.058	0.068	0.959	0.044
SBW		0.035	0.053	0.064	0.943	0.044
NAIVE		0.082	0.047	0.095	0.530	0.103
GLM	(D)	-0.015	0.119	0.120	0.913	-0.019
LASSO		-0.001	0.081	0.081	0.963	-0.002
npCBPS		0.023	0.096	0.098	0.955	0.028
CAL-ET		0.023	0.096	0.099	0.966	0.029
SBW		0.028	0.075	0.080	0.978	0.034
NAIVE		0.086	0.047	0.098	0.520	0.108
GLM	(E)	-0.020	0.106	0.108	0.915	-0.025
LASSO		0.007	0.073	0.073	0.961	0.009
npCBPS		0.046	0.097	0.108	0.922	0.057
CAL-ET		0.053	0.087	0.102	0.966	0.066
SBW		0.052	0.072	0.089	0.949	0.065
NAIVE		0.089	0.048	0.102	0.467	0.112
GLM	(F)	-0.004	0.110	0.110	0.931	-0.004
LASSO		0.015	0.074	0.075	0.970	0.019
npCBPS		0.020	0.098	0.100	0.950	0.025
CAL-ET		0.026	0.091	0.095	0.968	0.033
SBW		0.031	0.073	0.079	0.968	0.038
NAIVE		0.103	0.048	0.113	0.382	0.128
GLM	(G)	-0.040	0.064	0.076	0.906	-0.051
LASSO		0.003	0.047	0.047	0.964	0.004
npCBPS		0.034	0.066	0.074	0.935	0.042
CAL-ET		0.048	0.060	0.077	0.941	0.060
SBW		0.050	0.057	0.076	0.920	0.063
NAIVE		0.094	0.047	0.105	0.449	0.118

Table S3: Poor overlap ($k = 3$). Results of 1000 simulation replicates for estimators of MHR_{ATT} for different DGPs (A) - (G). Bias, Monte Carlo standard deviation (SD), root mean-squared error (RMSE), empirical coverage probability of 95% confidence intervals (Coverage and relative bias (Rel.Bias). Sample size is $n = 1500$, true $\text{MHR}_{ATT} = 0.8$ and no censoring ($\pi = 0$). In scenario (A) there is no model misspecification.

Method	Scenario	Bias	SD	RMSE	Coverage	Rel. Bias
GLM		0.035	0.203	0.206	0.906	0.044
LASSO		0.028	0.114	0.118	0.953	0.035
npCBPS	(A)	0.021	0.129	0.130	0.942	0.026
CAL-ET		0.022	0.159	0.160	0.923	0.027
SBW		0.026	0.096	0.099	0.961	0.032
NAIVE		0.104	0.047	0.114	0.346	0.130
GLM		0.002	0.197	0.197	0.898	0.002
LASSO		0.028	0.094	0.098	0.959	0.034
npCBPS	(B)	0.053	0.117	0.128	0.885	0.066
CAL-ET		0.059	0.119	0.133	0.963	0.074
SBW		0.059	0.083	0.102	0.953	0.073
NAIVE		0.107	0.048	0.117	0.341	0.134
GLM		-0.020	0.057	0.061	0.952	-0.025
LASSO		0.003	0.048	0.048	0.970	0.004
npCBPS	(C)	0.035	0.071	0.079	0.943	0.043
CAL-ET		0.044	0.065	0.078	0.958	0.055
SBW		0.045	0.059	0.074	0.932	0.056
NAIVE		0.088	0.048	0.100	0.480	0.110
GLM		0.002	0.218	0.218	0.863	0.002
LASSO		0.008	0.103	0.103	0.944	0.010
npCBPS	(D)	0.039	0.126	0.132	0.928	0.048
CAL-ET		0.046	0.192	0.198	0.932	0.058
SBW		0.050	0.100	0.112	0.960	0.063
NAIVE		0.096	0.046	0.107	0.417	0.121
GLM		-0.037	0.166	0.171	0.837	-0.046
LASSO		0.021	0.090	0.093	0.961	0.027
npCBPS	(E)	0.070	0.134	0.152	0.860	0.088
CAL-ET		0.086	0.134	0.160	0.954	0.108
SBW		0.084	0.094	0.126	0.912	0.105
NAIVE		0.094	0.050	0.106	0.454	0.118
GLM		0.009	0.190	0.190	0.887	0.011
LASSO		0.024	0.088	0.091	0.957	0.030
npCBPS	(F)	0.044	0.115	0.123	0.932	0.054
CAL-ET		0.045	0.166	0.172	0.950	0.057
SBW		0.045	0.094	0.104	0.964	0.056
NAIVE		0.111	0.049	0.121	0.317	0.139
GLM		-0.067	0.085	0.108	0.828	-0.084
LASSO		0.004	0.047	0.047	0.972	0.005
npCBPS	(G)	0.049	0.072	0.087	0.933	0.061
CAL-ET		0.062	0.068	0.092	0.944	0.077
SBW		0.063	0.062	0.088	0.903	0.079
NAIVE		0.099	0.046	0.109	0.404	0.124

Table S4: Censoring rate $\pi = 0.1$ Results of 1000 simulation replicates for estimators of MHR_{ATT} for different DGPs (A) - (G). Bias, Monte Carlo standard deviation (SD), root mean-squared error (RMSE), empirical coverage probability of 95% confidence intervals (Coverage and relative bias (Rel.Bias). Sample size is $n = 1500$, true $\text{MHR}_{ATT} = 0.8$ and good overlap $(k = 1)$. In scenario (A) there is no model misspecification.

Method	Scenario	Bias	SD	RMSE	Coverage	Rel. Bias
GLM	(A)	0.003	0.051	0.051	0.973	0.004
LASSO		0.004	0.050	0.050	0.974	0.005
npCBPS		-0.007	0.054	0.054	0.972	-0.008
CAL-ET		0.003	0.049	0.049	0.977	0.004
SBW		0.004	0.047	0.047	0.983	0.005
NAIVE		0.062	0.048	0.078	0.723	0.077
GLM	(B)	0.005	0.051	0.052	0.971	0.007
LASSO		0.007	0.050	0.051	0.970	0.009
npCBPS		0.006	0.059	0.059	0.959	0.007
CAL-ET		0.014	0.050	0.052	0.969	0.017
SBW		0.014	0.048	0.050	0.969	0.018
NAIVE		0.068	0.049	0.084	0.679	0.084
GLM	(C)	-0.001	0.044	0.044	0.970	-0.002
LASSO		0.001	0.043	0.043	0.972	0.001
npCBPS		0.009	0.046	0.047	0.981	0.011
CAL-ET		0.018	0.047	0.050	0.966	0.022
SBW		0.018	0.046	0.050	0.962	0.023
NAIVE		0.064	0.048	0.080	0.707	0.080
GLM	(D)	-0.006	0.054	0.055	0.971	-0.007
LASSO		-0.003	0.052	0.052	0.967	-0.004
npCBPS		0.003	0.060	0.061	0.971	0.004
CAL-ET		0.008	0.052	0.053	0.979	0.010
SBW		0.010	0.049	0.050	0.976	0.013
NAIVE		0.064	0.048	0.080	0.722	0.080
GLM	(E)	-0.001	0.055	0.055	0.961	-0.002
LASSO		0.004	0.052	0.052	0.966	0.004
npCBPS		0.016	0.063	0.065	0.958	0.020
CAL-ET		0.022	0.053	0.058	0.965	0.027
SBW		0.023	0.049	0.054	0.968	0.028
NAIVE		0.067	0.048	0.082	0.699	0.083
GLM	(F)	-0.004	0.057	0.057	0.953	-0.005
LASSO		-0.000	0.053	0.053	0.963	-0.000
npCBPS		-0.003	0.057	0.057	0.974	-0.004
CAL-ET		0.008	0.054	0.054	0.963	0.010
SBW		0.011	0.052	0.053	0.963	0.014
NAIVE		0.070	0.049	0.086	0.661	0.088
GLM	(G)	-0.010	0.046	0.048	0.971	-0.012
LASSO		-0.001	0.045	0.045	0.973	-0.002
npCBPS		0.017	0.050	0.053	0.962	0.022
CAL-ET		0.026	0.048	0.055	0.955	0.033
SBW		0.028	0.048	0.055	0.948	0.035
NAIVE		0.072	0.050	0.088	0.651	0.090

Table S5: Censoring rate $\pi = 0.2$ Results of 1000 simulation replicates for estimators of MHR_{ATT} for different DGPs (A) - (G). Bias, Monte Carlo standard deviation (SD), root mean-squared error (RMSE), empirical coverage probability of 95% confidence intervals (Coverage and relative bias (Rel.Bias). Sample size is $n = 1500$, true $\text{MHR}_{ATT} = 0.8$ and good overlap ($k = 1$). In scenario (A) there is no model misspecification.

Method	Scenario	Bias	SD	RMSE	Coverage	Rel. Bias
GLM	(A)	0.000	0.054	0.054	0.968	0.000
LASSO		0.001	0.053	0.053	0.971	0.002
npCBPS		-0.008	0.058	0.058	0.971	-0.010
CAL-ET		-0.000	0.053	0.053	0.976	-0.000
SBW		0.000	0.051	0.051	0.976	0.001
NAIVE		0.061	0.048	0.078	0.775	0.076
GLM	(B)	0.002	0.054	0.054	0.963	0.002
LASSO		0.003	0.052	0.052	0.964	0.004
npCBPS		0.002	0.061	0.061	0.972	0.003
CAL-ET		0.010	0.052	0.053	0.979	0.013
SBW		0.011	0.050	0.051	0.973	0.013
NAIVE		0.064	0.051	0.082	0.737	0.081
GLM	(C)	-0.007	0.045	0.046	0.975	-0.009
LASSO		-0.005	0.044	0.044	0.973	-0.006
npCBPS		0.003	0.049	0.049	0.979	0.004
CAL-ET		0.012	0.046	0.048	0.975	0.015
SBW		0.013	0.045	0.047	0.977	0.016
NAIVE		0.058	0.049	0.076	0.777	0.073
GLM	(D)	-0.009	0.062	0.062	0.954	-0.012
LASSO		-0.006	0.059	0.059	0.960	-0.007
npCBPS		-0.000	0.069	0.069	0.977	-0.000
CAL-ET		0.007	0.059	0.060	0.970	0.009
SBW		0.007	0.056	0.056	0.969	0.009
NAIVE		0.059	0.050	0.077	0.770	0.074
GLM	(E)	-0.006	0.057	0.058	0.968	-0.008
LASSO		-0.002	0.054	0.054	0.972	-0.003
npCBPS		0.008	0.067	0.067	0.960	0.010
CAL-ET		0.016	0.056	0.058	0.974	0.021
SBW		0.018	0.052	0.056	0.967	0.022
NAIVE		0.060	0.050	0.078	0.766	0.075
GLM	(F)	-0.003	0.058	0.058	0.973	-0.003
LASSO		0.001	0.054	0.054	0.973	0.001
npCBPS		-0.001	0.064	0.064	0.974	-0.001
CAL-ET		0.010	0.056	0.057	0.972	0.012
SBW		0.011	0.052	0.053	0.978	0.014
NAIVE		0.071	0.051	0.088	0.710	0.089
GLM	(G)	-0.019	0.051	0.055	0.947	-0.024
LASSO		-0.010	0.049	0.050	0.955	-0.013
npCBPS		0.006	0.052	0.052	0.971	0.008
CAL-ET		0.017	0.051	0.054	0.972	0.021
SBW		0.019	0.051	0.054	0.963	0.024
NAIVE		0.066	0.051	0.084	0.721	0.082

Table S6: Censoring rate $\pi = 0.3$ Results of 1000 simulation replicates for estimators of MHR_{ATT} for different DGPs (A) - (G). Bias, Monte Carlo standard deviation (SD), root mean-squared error (RMSE), empirical coverage probability of 95% confidence intervals (Coverage and relative bias (Rel.Bias). Sample size is $n = 1500$, true $\text{MHR}_{ATT} = 0.8$ and good overlap ($k = 1$). In scenario (A) there is no model misspecification.

Method	Scenario	Bias	SD	RMSE	Coverage	Rel. Bias
GLM	(A)	-0.002	0.058	0.058	0.972	-0.002
LASSO		-0.001	0.056	0.056	0.977	-0.001
npCBPS		-0.012	0.063	0.064	0.975	-0.015
CAL-ET		-0.002	0.055	0.055	0.976	-0.003
SBW		-0.002	0.052	0.052	0.979	-0.003
NAIVE		0.057	0.052	0.077	0.812	0.072
GLM	(B)	-0.003	0.056	0.056	0.962	-0.004
LASSO		-0.002	0.055	0.055	0.965	-0.002
npCBPS		-0.002	0.065	0.065	0.972	-0.003
CAL-ET		0.005	0.055	0.055	0.972	0.006
SBW		0.005	0.052	0.052	0.972	0.007
NAIVE		0.060	0.053	0.080	0.801	0.075
GLM	(C)	-0.011	0.049	0.050	0.972	-0.013
LASSO		-0.008	0.048	0.049	0.974	-0.010
npCBPS		0.001	0.052	0.052	0.974	0.001
CAL-ET		0.009	0.052	0.052	0.974	0.012
SBW		0.010	0.050	0.051	0.976	0.013
NAIVE		0.056	0.053	0.077	0.822	0.070
GLM	(D)	-0.011	0.066	0.067	0.961	-0.014
LASSO		-0.008	0.062	0.062	0.956	-0.010
npCBPS		-0.004	0.075	0.075	0.963	-0.005
CAL-ET		0.003	0.062	0.063	0.976	0.004
SBW		0.004	0.057	0.058	0.975	0.005
NAIVE		0.057	0.054	0.079	0.805	0.072
GLM	(E)	-0.014	0.062	0.063	0.955	-0.017
LASSO		-0.010	0.058	0.059	0.959	-0.012
npCBPS		0.003	0.076	0.076	0.958	0.004
CAL-ET		0.010	0.061	0.062	0.972	0.012
SBW		0.011	0.057	0.058	0.966	0.014
NAIVE		0.055	0.054	0.077	0.813	0.068
GLM	(F)	-0.009	0.060	0.061	0.969	-0.012
LASSO		-0.005	0.057	0.057	0.971	-0.007
npCBPS		-0.009	0.063	0.063	0.971	-0.011
CAL-ET		0.003	0.058	0.058	0.979	0.004
SBW		0.005	0.056	0.056	0.974	0.006
NAIVE		0.064	0.052	0.082	0.781	0.080
GLM	(G)	-0.023	0.053	0.058	0.948	-0.029
LASSO		-0.014	0.051	0.052	0.961	-0.017
npCBPS		0.004	0.057	0.058	0.977	0.005
CAL-ET		0.014	0.054	0.056	0.974	0.017
SBW		0.015	0.053	0.055	0.974	0.019
NAIVE		0.062	0.053	0.081	0.780	0.077

Table S7: Censoring rate $\pi = 0.4$ Results of 1000 simulation replicates for estimators of MHR_{ATT} for different DGPs (A) - (G). Bias, Monte Carlo standard deviation (SD), root mean-squared error (RMSE), empirical coverage probability of 95% confidence intervals (Coverage and relative bias (Rel.Bias). Sample size is $n = 1500$, true $\text{MHR}_{ATT} = 0.8$ and good overlap ($k = 1$). In scenario (A) there is no model misspecification.

Method	Scenario	Bias	SD	RMSE	Coverage	Rel. Bias
GLM	(A)	-0.004	0.062	0.062	0.969	-0.005
LASSO		-0.003	0.060	0.061	0.973	-0.004
npCBPS		-0.013	0.068	0.069	0.966	-0.016
CAL-ET		-0.004	0.060	0.060	0.969	-0.005
SBW		-0.004	0.057	0.057	0.978	-0.004
NAIVE		0.059	0.060	0.084	0.826	0.074
GLM	(B)	-0.009	0.060	0.061	0.966	-0.012
LASSO		-0.008	0.059	0.060	0.967	-0.010
npCBPS		-0.009	0.069	0.069	0.975	-0.011
CAL-ET		-0.001	0.059	0.059	0.974	-0.001
SBW		-0.001	0.056	0.056	0.972	-0.001
NAIVE		0.055	0.058	0.080	0.846	0.069
GLM	(C)	-0.019	0.053	0.057	0.952	-0.024
LASSO		-0.017	0.052	0.055	0.954	-0.022
npCBPS		-0.008	0.060	0.061	0.967	-0.010
CAL-ET		0.001	0.056	0.056	0.974	0.001
SBW		0.001	0.054	0.054	0.975	0.001
NAIVE		0.048	0.056	0.074	0.866	0.060
GLM	(D)	-0.017	0.069	0.071	0.940	-0.022
LASSO		-0.014	0.066	0.067	0.942	-0.018
npCBPS		-0.011	0.078	0.079	0.960	-0.013
CAL-ET		-0.003	0.066	0.066	0.961	-0.004
SBW		-0.001	0.062	0.062	0.964	-0.001
NAIVE		0.052	0.057	0.077	0.860	0.064
GLM	(E)	-0.019	0.066	0.068	0.952	-0.024
LASSO		-0.015	0.062	0.064	0.956	-0.019
npCBPS		0.001	0.081	0.081	0.956	0.002
CAL-ET		0.005	0.064	0.064	0.961	0.006
SBW		0.005	0.060	0.060	0.971	0.007
NAIVE		0.052	0.057	0.077	0.854	0.065
GLM	(F)	-0.015	0.068	0.070	0.949	-0.018
LASSO		-0.011	0.064	0.064	0.954	-0.014
npCBPS		-0.011	0.075	0.076	0.963	-0.014
CAL-ET		-0.002	0.065	0.065	0.961	-0.002
SBW		-0.001	0.062	0.062	0.968	-0.001
NAIVE		0.060	0.059	0.084	0.818	0.074
GLM	(G)	-0.034	0.056	0.066	0.931	-0.043
LASSO		-0.025	0.054	0.059	0.952	-0.031
npCBPS		-0.006	0.061	0.062	0.966	-0.008
CAL-ET		0.002	0.058	0.058	0.972	0.003
SBW		0.004	0.056	0.056	0.973	0.005
NAIVE		0.054	0.055	0.077	0.861	0.067

Table S8: Censoring rate $\pi = 0.5$ Results of 1000 simulation replicates for estimators of MHR_{ATT} for different DGPs (A) - (G). Bias, Monte Carlo standard deviation (SD), root mean-squared error (RMSE), empirical coverage probability of 95% confidence intervals (Coverage and relative bias (Rel.Bias). Sample size is $n = 1500$, true $\text{MHR}_{ATT} = 0.8$ and good overlap ($k = 1$). In scenario (A) there is no model misspecification.

Method	Scenario	Bias	SD	RMSE	Coverage	Rel. Bias
GLM	(A)	-0.014	0.067	0.068	0.968	-0.018
LASSO		-0.013	0.065	0.066	0.972	-0.016
npCBPS		-0.022	0.075	0.078	0.956	-0.028
CAL-ET		-0.014	0.065	0.067	0.965	-0.018
SBW		-0.013	0.062	0.063	0.972	-0.016
NAIVE		0.051	0.060	0.079	0.882	0.064
GLM	(B)	-0.016	0.066	0.068	0.959	-0.020
LASSO		-0.015	0.064	0.066	0.962	-0.019
npCBPS		-0.015	0.076	0.077	0.970	-0.019
CAL-ET		-0.008	0.065	0.065	0.971	-0.010
SBW		-0.008	0.062	0.062	0.971	-0.010
NAIVE		0.048	0.062	0.078	0.883	0.060
GLM	(C)	-0.030	0.059	0.066	0.947	-0.037
LASSO		-0.028	0.058	0.064	0.947	-0.034
npCBPS		-0.018	0.067	0.069	0.960	-0.022
CAL-ET		-0.010	0.061	0.062	0.972	-0.012
SBW		-0.009	0.059	0.060	0.971	-0.011
NAIVE		0.040	0.062	0.074	0.897	0.049
GLM	(D)	-0.022	0.078	0.081	0.938	-0.028
LASSO		-0.019	0.073	0.075	0.946	-0.023
npCBPS		-0.019	0.089	0.091	0.949	-0.023
CAL-ET		-0.008	0.075	0.075	0.960	-0.010
SBW		-0.008	0.068	0.068	0.964	-0.010
NAIVE		0.049	0.062	0.079	0.898	0.061
GLM	(E)	-0.025	0.075	0.079	0.929	-0.031
LASSO		-0.021	0.071	0.074	0.941	-0.026
npCBPS		-0.010	0.090	0.090	0.957	-0.012
CAL-ET		-0.002	0.072	0.072	0.967	-0.002
SBW		-0.003	0.067	0.067	0.967	-0.003
NAIVE		0.042	0.061	0.074	0.896	0.052
GLM	(F)	-0.017	0.072	0.074	0.959	-0.021
LASSO		-0.013	0.068	0.069	0.967	-0.016
npCBPS		-0.012	0.080	0.081	0.963	-0.015
CAL-ET		-0.005	0.068	0.068	0.979	-0.006
SBW		-0.005	0.063	0.064	0.981	-0.006
NAIVE		0.060	0.061	0.085	0.866	0.075
GLM	(G)	-0.044	0.065	0.078	0.902	-0.055
LASSO		-0.035	0.061	0.070	0.922	-0.043
npCBPS		-0.017	0.067	0.070	0.963	-0.022
CAL-ET		-0.006	0.065	0.065	0.968	-0.008
SBW		-0.004	0.062	0.063	0.972	-0.005
NAIVE		0.045	0.062	0.076	0.892	0.056

Table S9: Overspecification. Results of 1000 simulation replicates for estimators of MHR_{ATT} for different DGPs (A) - (G). Bias, Monte Carlo standard deviation (SD), root mean-squared error (RMSE), empirical coverage probability of 95% confidence intervals (Coverage and relative bias (Rel.Bias). Sample size is $n = 1500$, true $\text{MHR}_{ATT} = 0.80$ no censoring ($\pi = 0$) and good overlap ($k = 1$). In scenario (A) there is no model misspecification.

Method	Scenario	Bias	SD	RMSE	Coverage	Rel. Bias
GLM	(A)	0.005	0.050	0.051	0.981	0.007
LASSO		0.006	0.047	0.048	0.980	0.008
npCBPS		–	–	–	–	–
CAL-ET		0.005	0.049	0.050	0.979	0.007
SBW		0.007	0.046	0.047	0.977	0.009
NAIVE		0.064	0.045	0.079	0.691	0.080
GLM	(B)	0.006	0.052	0.053	0.972	0.007
LASSO		0.007	0.049	0.050	0.965	0.008
npCBPS		–	–	–	–	–
CAL-ET		0.007	0.051	0.052	0.972	0.009
SBW		0.010	0.047	0.048	0.975	0.012
NAIVE		0.074	0.047	0.088	0.622	0.093
GLM	(C)	0.004	0.094	0.094	0.943	0.005
LASSO		0.004	0.061	0.061	0.956	0.004
npCBPS		–	–	–	–	–
CAL-ET		0.006	0.074	0.074	0.965	0.007
SBW		0.009	0.061	0.061	0.969	0.011
NAIVE		0.065	0.046	0.080	0.682	0.081
GLM	(D)	0.005	0.059	0.059	0.967	0.007
LASSO		0.005	0.053	0.053	0.967	0.007
npCBPS		–	–	–	–	–
CAL-ET		0.005	0.057	0.057	0.973	0.006
SBW		0.008	0.051	0.052	0.976	0.010
NAIVE		0.065	0.046	0.079	0.685	0.081
GLM	(E)	0.007	0.058	0.059	0.968	0.009
LASSO		0.007	0.052	0.053	0.976	0.008
npCBPS		–	–	–	–	–
CAL-ET		0.008	0.057	0.058	0.969	0.010
SBW		0.010	0.051	0.052	0.971	0.013
NAIVE		0.070	0.047	0.084	0.647	0.087
GLM	(F)	0.006	0.059	0.059	0.954	0.007
LASSO		0.008	0.053	0.054	0.962	0.010
npCBPS		–	–	–	–	–
CAL-ET		0.006	0.056	0.056	0.973	0.007
SBW		0.007	0.050	0.051	0.974	0.008
NAIVE		0.074	0.047	0.087	0.637	0.092
GLM	(G)	0.011	0.105	0.106	0.949	0.014
LASSO		0.009	0.062	0.063	0.956	0.011
npCBPS		–	–	–	–	–
CAL-ET		0.009	0.080	0.081	0.960	0.011
SBW		0.014	0.062	0.063	0.969	0.018
NAIVE		0.076	0.048	0.090	0.587	0.096

Table S10: Computational time finding weights with different methods. Median time in seconds based on code running 10 times using the R function `microbenchmark` [3]. Scenario (A), $k = 1$, $n = 1500$ and gurobi as SBW solver. The computations were run on a MacBook Pro (2018) with 2,7 GHz Intel Core i7 processor, and 16 GB 2133 MHz LPDDR3 memory.

Method	$\mathbf{X}_{\text{small}}$	$\mathbf{X}_{\text{large}}$
GLM	0.005	0.031
LASSO	11.144	20.118
npCBPS	21.881	–
CAL-ET	0.354	4.309
SBW	11.889	39.674

C S3 Appendix: Case study results

Figure S6: Estimated overlap in the case study data. The propensity score is estimated using GLM with $\mathbf{X}_{\text{large}}$.

References

[1] Wan, F. Simulating survival data with predefined censoring rates for proportional hazards models. *Statistics In Medicine.* **36** pp. 838-854 (2017)

[2] Silverman, B. Density Estimation for Statistics and Data Analysis. (Chapman & Hall,1986)

[3] Mersmann, O. microbenchmark: Accurate Timing Functions. (2021), https://CRAN.R-project.org/package=microbenchmark, R package version 1.4.9
