# Supplementary material for: Performance of modeling and balancing approach methods when using weights to estimate treatment effects in observational time-to-event settings
Source: PLoS One. 2023 Dec 7;18(12):e0289316. doi: 10.1371/journal.pone.0289316 (PMC10703278; doi:10.1371/journal.pone.0289316)
Supplement: Attachment — Submitted filename: Response to Reviewers.pdf [file pone.0289316.s008.pdf]

Response to reviewers, PONE-D-23-04363

Dear Editor,
We are grateful for the opportunity to revise our manuscript entitled “Performance of modeling and balancing approach methods when using weights to estimate treatment effects in observational time-to-event settings” in light of the editorial and reviewer comments. Below, we have provided a point-by-point response.

Academic editor's comments

1. Please ensure that your manuscript meets PLOS ONE's style requirements, including those for file naming.

Response:
We have ensured that our manuscript meets PLOS ONE's style requirements.

2. Please note that PLOS ONE has specific guidelines on code sharing for submissions in which author-generated code underpins the findings in the manuscript. In these cases, all author-generated code must be made available without restrictions upon publication of the work. Please review our guidelines at https://journals.plos.org/plosone/s/materials-and-software-sharing#loc-sharing-code and ensure that your code is shared in a way that follows best practice and facilitates reproducibility and reuse.

Response:
All author-generated code has been made available on GitHub. We have provided the following amended data availability statement:

The code used to generate the data in the Monte Carlo simulation is available on the github repository: https://github.com/Wangbarros/Modeling_vs_Balancing_Time_to_Event. The data analyzed in the case study consist of third party data from Riksstroke and according to Swedish legislation (https:// etikprovningsmyndigheten.se/for-forskare/vadsager-lagen/) data cannot be made available for use beyond what has been approved by the ethical review authority. Therefore, the data cannot be made publicly available. Data may be made available from Riksstroke (contact via riksstroke@regionvasterbotten.se) upon reasonable request by researchers who meet the criteria for access to confidential data according to Swedish laws and regulations.

3. Please ensure that you have specified (1) whether consent was informed and (2) what type you obtained (for instance, written or verbal, and if verbal, how it was documented and witnessed). If your study included minors, state whether you obtained consent from parents or guardians. If the need for consent was waived by the ethics committee, please include this information.

Response:
We have added the following sentence to the ethics statement in the "Materials and Methods" section:

"Patients and next of kin are informed about the registration and aim of the Riksstroke-register and their right to decline participation (opt-out consent)."

4. Thank you for stating in your Funding Statement: "This work was supported by the Swedish Research Council (Dnr: 2018–01610). The funders had no role in study design, data collection and analysis, decision to publish, or preparation of the manuscript." Please provide an amended statement that declares *all* the funding or sources of support (whether external or internal to your organization) received during this study, as detailed online in our guide for authors at http://journals.plos.org/plosone/s/submit-now. Please also include the statement "There was no additional external funding received for this study." in your updated Funding Statement. Please include your amended Funding Statement within your cover letter. We will change the online submission form on your behalf.

Response:
We have provided the following amended Funding Statement:

"This work was supported by the Swedish Research Council (Dnr: 2018–01610; recipient JH). The funders had no role in study design, data collection and analysis, decision to publish, or preparation of the manuscript. There was no additional external funding received for this study."

5. Thank you for stating the following in the Acknowledgments Section of your manuscript: "The authors are grateful to Associate professor Maria Josefsson for helpful and 376 constructive comments. This work was supported by the Swedish Research Council 377 (Dnr: 2018–01610)."
We note that you have provided additional information within the Acknowledgements Section that is not currently declared in your Funding Statement. Please note that funding information should not appear in the Acknowledgments section or other areas of your manuscript. We will only publish funding information present in the Funding Statement section of the online submission form.

Please remove any funding-related text from the manuscript and let us know how you would like to update your Funding Statement. Currently, your Funding Statement reads as follows:
"This work was supported by the Swedish Research Council (Dnr: 2018–01610).
The funders had no role in study design, data collection and analysis, decision to publish, or preparation of the manuscript." Please include your amended statements within your cover letter; we will change the online submission form on your behalf.

Response:
We have amended the Acknowledgements Section in the manuscript to:

"The authors are grateful to Associate Professor Maria Josefsson for helpful and constructive comments. "

The funding statement has been changed as described in the response to comment 4 above.

6. Your ethics statement should only appear in the Methods section of your manuscript. If your ethics statement is written in any section besides the Methods, please move it to the Methods section and delete it from any other section. Please ensure that your ethics statement is included in your manuscript, as the ethics statement entered into the online submission form will not be published alongside your manuscript.

Response:
We have now included the following ethics statement in the "Materials and methods" section of the manuscript:

"Statistical method development for fair comparisons of stroke care and outcome was part of the EqualStroke-project, approved by the Ethical Review Board in Umeå (Dnr: 2012-321-31M, 2014-76-32M). Patients and next of kin are informed about the registration and aim of the Riksstroke-register and their right to decline participation (opt-out consent)."

7. Please include captions for your Supporting Information files at the end of your manuscript, and update any in-text citations to match accordingly. Please see our Supporting Information guidelines for more information: http://journals.plos.org/plosone/s/supporting-information.

Response:
We have included captions for the Supporting Information files at the end of the manuscript and updated in-text citations.

Reviewer #1

Comment
The paper is good. The presentation of the article is also good. The manuscript is technically sound, and do the data support the conclusions. The statistical analysis has been performed appropriately and rigorously. The authors have made all data underlying the findings in their manuscript fully available. The manuscript is not presented in an intelligible fashion and written in standard English.

Response:
We have engaged a proofreading agency and made changes to the text according to the proofreader's suggestions.

Reviewer #2

Comment
This paper investigates the finite sample properties of different weighting methods when these are used to estimate population level treatment effects using survival data. It is a little research point, however I have some comments to improve the overall look of the paper: the authors should

1. Review the paper in general linguistically

Response:
We have engaged a proofreading agency and made changes to the text according to the proofreader's suggestions.

2. Adding recent studies that dealt with the same point

Response:
We have reviewed recent literature dealing with similar research questions as is the focus of our manuscript and added a few more references and comments on similar work (see citations 57 and 59)

3. Modify the abstract to clarify the purpose of the study, its importance, and the most important findings of their study.

Response:
We have modified the abstract and clarified the purpose, importance and most important findings of the study.

4. Rewrite the discussion section because it is poor, and clarify the advantages and disadvantages of each method. Explain the reason for preferring one over the other in light of the discussion of previous studies and research.

Response:
We have rewritten the discussion.

Reviewer #3

Comment
This article compares the modelling vs balancing approach to estimate weights for weighted causal effect estimators of binary exposures and time-to-event outcomes. The article contains sufficient details in the description of the methods, provides a comprehensive simulation study and applies several methods to a real data application. The article is also well written. I only have one major comment, and a few minor comments.

Major comment
The sum of the balancing weights is set to 1. In contrast, for estimating the ATT, the sum of the weights based on the propensity score for the control patients will tend to n1, the sample size of the treated patients. This discrepancy would seem to make the comparison between the balancing and modelling approach unfair, as the scaling factor for the weights has an impact on the estimation of the marginal causal hazard ratio in the treated patients. Please set the sum of the balancing weights to be equal to n1 when estimating the ATT.

Response:
When we use weights based on the propensity score, for estimating ATT, the sum of the weights for the treated patients is n1 (since each treated patient get weight = 1) and the sum of the weights for the control patient tend to n1 (as the reviewer points out). When we use balancing weights, for estimating ATT, the weights are scaled such that the sum of weights for treated patients is equal to 1 and the sum of the weights for the control patients is also set to 1. Changing the scaling such that both the sum of weights for the treated and control groups would be n1 instead of 1 would not make a difference. If it were the case that the balancing weights for treated summed to n1 then, we agree with the reviewer, that it would be inappropriate to set the sum of the balancing weights to 1.

To clarify this we have added the following sentence in the manuscript when discussing balancing approaches:

“It should be also noted that, for the estimation of ATT weights, all treated individuals receive the same weights, and the sum of the weights for the treated individuals will also be equal to 1.”

Minor comments

1. It is worth clarifying on page 8 that the use of the robust variance estimator for estimating the ATT characterized by the marginal hazard ratio was found to lead to conservative inference in simulations only, and that no theoretical justification has been provided. For instance the following article shows that the robust variance estimator can also lead to anti-conservative inference “On Variance of the Treatment Effect in the Treated When Estimated by Inverse Probability Weighting”

Response:
This has now been clarified and the amended sentience in the manuscript is:

“Although no theoretical justification has been provided, simulation results have indicated that this estimator slightly overestimates the variance, and results in somewhat conservative confidence intervals when estimating MHR_ATE and MHR_ATT with IPTW weights [46]. However, more recently the possibility of anti-conservative inference when estimating ATT with IPTW weights was shown [47]. “

2. On page 5, it is worth clarifying that entropy balancing is double robust with respect to a linear outcome regression model and logistic propensity score model. The current setting assumes a log-linear regression model for the hazard, and therefore does not fall in the same setting.

Response:
The sentence on double robustness of entropy balance has been amended to:

“EB was first proposed as a preprocessing method without considering how this preprocessing would impact any subsequent inference [19], but has since been shown to be doubly robust in settings where the outcome model is linear and the PS model is logistic [34].”

3. It is worth mentioning in the discussion that the overlap weights can also be used as an approach to create exact balance if the user is willing to change the target population to one where there is high overlap in the covariate distributions.

Response:
The following sentence has been included in the Discussion:

"It is also worth noting that if, in a poor overlap situation, one is willing to redefine the target population to a subpopulation with good overlap an alternative approach is to use overlap weights [58], which yield exact balance if estimated by logistic regression."
